# Robotic Beyond Total Mesorectal Excision With Synchronous Perineal Flap Reconstruction: Feasibility and Early Outcomes

**DOI:** 10.1002/rcs.70223

**Published:** 2026-10-10

**Authors:** Ahlam H. Alharbi, Muhammad R. Iqbal, Dolly M. Dowsett, Eleanor J. M. Rudge, Hassan I. Shah, Sandeep Kapur, Richard M. Haywood, Anais Rosich‐Medina, Irshad A. Shaikh

**Affiliations:** ^1^ Department of Surgery, Faculty of Medicine in Rabigh King Abdulaziz University Rabigh Saudi Arabia; ^2^ Mid and South Essex NHS Foundation Trust Basildon UK; ^3^ Sir Thomas Browne Academic Colorectal Unit Norfolk and Norwich University Hospital NHS Foundation Trust Norwich UK; ^4^ Sheffield Teaching Hospital NHS Foundation Trust Sheffield UK; ^5^ Department of Plastic Surgery Norfolk and Norwich University Hospital NHS Foundation Trust Norwich UK; ^6^ Department of Anatomy Norwich Medical School, University of East Anglia Norwich UK; ^7^ Sir Thomas Browne Academic Colorectal Unit, Norfolk and Norwich University Hospital NHS Foundation Trust, University of East Anglia Norwich UK

**Keywords:** beyond total mesorectal excision (bTME), case series, gracilis flap reconstruction, perineal reconstruction, robotic surgery

## Abstract

**Background:**

Advanced rectal and anal malignancies often require dissections beyond total mesorectal planes for improved oncological outcomes. While minimally invasive techniques facilitate this approach, they result in larger perineal defects, creating reconstructive challenges. Robotic‐assisted surgery enhances precision, visualisation, and ergonomics, potentially optimising outcomes. However, data on robotics beyond total mesorectal excision (bTME) remain limited. This study evaluated robotic bTME and synchronous gracilis or PAP (profunda artery perforator) flap reconstruction.

**Methods:**

This retrospective case series reviewed 10 patients with locally advanced or recurrent anorectal cancer who underwent elective robotic bTME with synchronous perineal gracilis or PAP flap reconstruction.

**Results:**

Median operative time was 465 minutes. Gracilis flap harvest was concurrent. No major flap‐related complications or reoperations. R0 resection was achieved in 80%. At 1‐year, local recurrence was 0%, distant metastases 30%, and disease‐free survival 70%.

**Conclusion:**

Robotics bTME with synchronous gracilis or PAP flap reconstruction is feasible, efficient, and oncologically safe.

## 1 Introduction

Colorectal cancer (CRC) is the third most common cancer worldwide, with rectal cancer representing a significant proportion [1]. Despite the advances in screening and treatment, 18%–20% of rectal cancers are presented as locally advanced, requiring extended resection beyond the total mesorectal plane to achieve oncological clearance (R0) [2]. R0 resection yields a 5‐year survival rate of approximately 60% [3].

Management of locally advanced or recurrent ano‐rectal cancer often necessitates extensive resections, such as beyond Total Mesorectal Excision (bTME) or Extralevator Abdominoperineal Excision (ELAPE), to achieve clear oncological margins. bTME involves resection beyond the standard mesorectal envelope to include adjacent structures, such as the pelvic sidewall, presacral fascia, or levator muscles, when oncologically necessary to achieve R0 resection. Robotic assisted bTME enhances precision in deep pelvic dissection, facilitating meticulous resection while preserving critical neurovascular structures [4]. Minimally invasive approaches have demonstrated oncological safety [5, 6], but anatomical challenges, high BMI and prior pelvic surgery or irradiation often necessitate open surgery.

Robotic surgery is increasingly adopted in colorectal practice owing to its benefits in reducing length of stay, promoting early recovery and decreasing complications [7]. Shin et al. first reported robotic pelvic exenteration in 2014 [8]. Robotic‐assisted bTME offers enhanced precision and ergonomics via wristed instruments and a stable operator‐controlled 3D camera, especially in complex or previously treated cases [9]. Despite these advances, extensive surgery is associated with significant perioperative morbidity and mortality (27%–86%) [10]. Large perineal defects add further morbidity. Reconstruction techniques such as omental flaps, mesh, and myocutaneous flaps show variable success [11, 12]. These multispecialty procedures, involving colorectal, plastic, and urology teams sequentially, also prolong operative time.

Although robotic bTME and flap reconstruction have been described separately, their combined use in a single coordinated procedure is underreported. This series presents our experience with simultaneous robotic bTME and perineal flap reconstruction, focussing on operative efficiency, complication rates, and oncological outcomes.

## 2 Material and Methods

### 2.1 Study Design and Patients

This retrospective case series included consecutive adult patients with primary or recurrent locally advanced anorectal cancer who underwent robotic bTME (pelvic exenteration or ELAPE) with simultaneous perineal flap reconstruction between July 2020 and March 2024. Patients undergoing standard robotic TME or abdominoperineal resection (APR) without flap reconstruction were excluded. Due to the rarity of this combined procedure, no formal sample size calculation was performed. All eligible patients within the study period were included to maximise available data and reduce selection bias.

### 2.2 Data Collection

Data were extracted from a prospectively maintained database using secure electronic records. Variables included demographics (age, sex, body mass index (BMI), American society of Anaesthesiologists (ASA) grade, co‐morbidities, cancer history), Tumour characteristics (histology, grade, TNM stage [AJCC 8th edition] [13], tumour distant from anal verge, and neoadjuvant chemoradiotherapy), and operative details (duration, blood loss, conversion to open surgery, analgesia use). Operative time was divided into robotic abdominal, flap harvest, urological, perineal dissection and flap reconstruction phases.

Postoperative outcomes included analgesia use, histology of specimen, resection margins (*R*0 > 1 mm), *R*1 ≤ 1 mm, R2 macroscopic residual), complications (Clavien‐Dindo classification) [14], length of stay, 30‐ and 90‐day readmissions, and 90‐day mortality. Follow‐up occurred at 30 days, 90 days, and 1 year. Oncological outcomes included local and systemic recurrence and overall survival.

Descriptive statistics were used. Continuous normally distributed variables reported as means ± standard deviations (SD) or medians with interquartile range (IQR) for skewed continuous variables and categorical variables as counts and percentages. No formal statistical comparisons were performed due to the small sample size.

### 2.3 Pathway

All cases were discussed at a multidisciplinary team (MDT) meeting. Pre‐operative assessments included colonoscopy and biopsy when indicated, CT of chest/abdomen/pelvis, and pelvic MRI. Rectal cancer patients with mesorectal fascial involvement or pelvic sidewall nodes received neoadjuvant long course chemoradiotherapy (LCCRT), whereas anal cancer patients received standard radiotherapy. Restaging occurred 6–10 weeks post‐treatment with CT, MRI and positron emission tomography (PET) scans. Patients were reviewed pre‐operatively by colorectal surgeons, specialist nurses, stoma nurses, plastic surgeons and urologists (if required). All procedures were performed using the Da Vinci X robotic system (Intuitive Surgical, Sunnyvale, CA, USA) by senior colorectal surgeons.

### 2.4 Surgical Procedure

Surgical team: Colorectal surgeons (I.S., S.K.) led the procedure; urological phase by O.A.K.; flap reconstruction by R.H., D.M., and A.R.M.

The operations included total pelvic exenteration, posterior pelvic exenteration and en bloc vaginal excision for anal cancers.

Preparation: Patients received preoperative antibiotics, thromboprophylaxis, stoma site marking, and a urinary catheterisation, and anaesthesia (general ± epidural/spinal). Patients positioned in modified Lloyd‐Davies with arms tucked ensuring the buttocks were exposed over the end of the table and sacrum elevated with a gel roll. The abdomen was prepped and draped from costal margin to symphysis pubis, the perineum was prepped after suture of the anus, the thighs were prepped circumferentially from the groin crease / buttock to the knee joint. Leg drapes and square drapes were secured to expose the whole thigh and cover the leg supports. Cystoscopy and double‐J stent insertion were performed if required (Figure 1).

**FIGURE 1:**
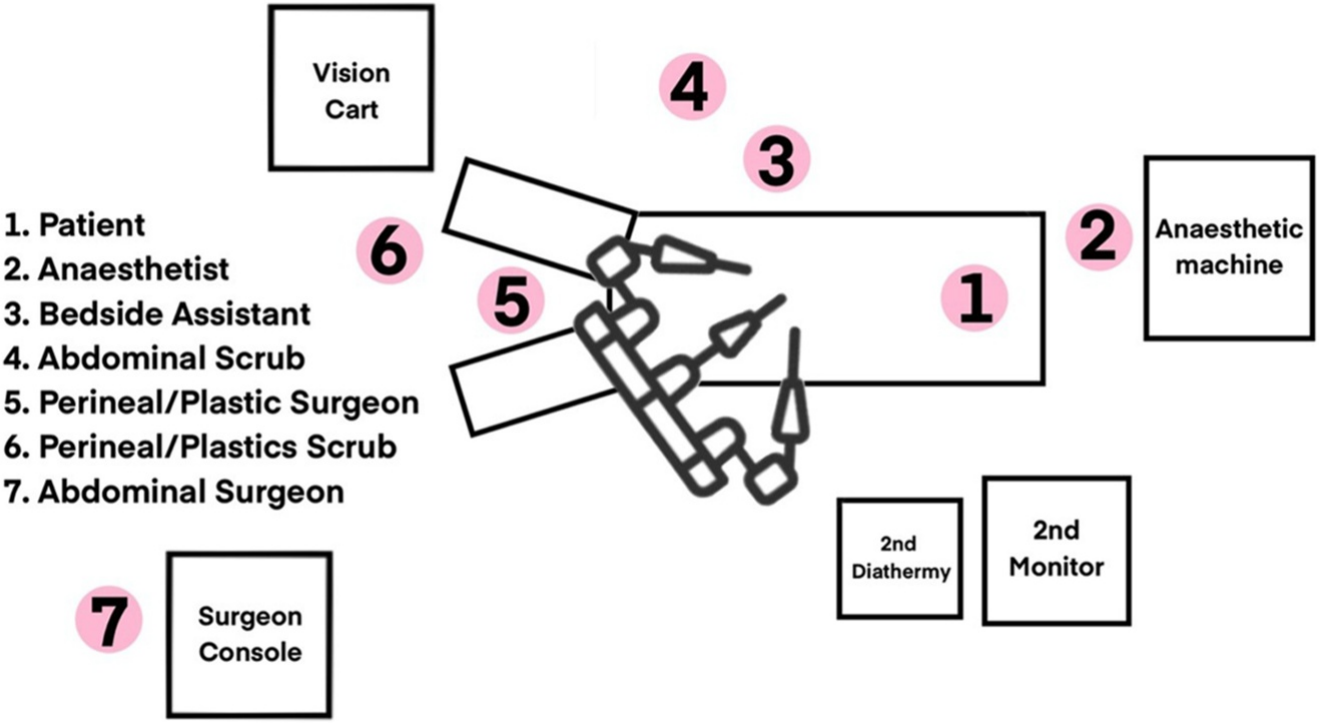
Diagram of the operating room setup for synchronous robotic surgery and flap reconstruction.

Robotic abdominal phase: A Hasson technique was used at the umbilicus to establish the pneumoperitoneum. Laparoscopy assessed metastatic disease and confirmed robotic feasibility. Four 8 mm robotic ports were placed obliquely, with a 5 mm Airseal assistant port (Figure 2). The patient was placed into 15° Trendelenburg and 10° left tilt. The robot was docked from the patient's left side. Dissection proceeded medially to laterally to mobilise the descending colon and splenic flexure. The inferior mesenteric artery and vein were ligated high using hem‐o‐locks. Entry into the lesser sac was achieved via dissection over the pancreas. The left ureter, gonadal vessels, and hypogastric nerves were preserved. Pelvic dissection commenced in the TME plane and extended based on tumour involvement.

Flap harvest: This was undertaken at the beginning of the operation simultaneously as the laparoscopy was being undertaken, robotic ports were being placed, robot being docked and abdominal resection commenced. The gracilis flaps are elevated as muscle only flaps through a 5‐8cm proximal incision over the flap pedicle and 2‐3 over the distal tendonous portion just proximal to the medial femoral condyle. If skin was required for vaginal wall reconstruction a Profunda Artery Perforator (PAP) flap was elevated and ipsilateral gracilis flap elevated through the same incision. Once a flap is elevated it is “parked” with a swab attached in a tunnel over the lateral edge of the posterior pubic ramus and the donor sites are closed [15]. If no training is undertaken the senior plastic surgery author can elevate and close one flap donor site before the robot is docked. Once the robot is docked, flap elevation can still be elevated with care taken to observe the movement of the robotic arms and careful liaison with the general team at the table. Bilateral gracilis flaps, PAP flaps elevation and gracilis donor sites closure were always complete before abdominal resection was complete. Real‐time intraoperative coordination was achieved through preoperative planning and verbal synchronisation among teams. Ports placement and patient positioning were optimised to allow concurrent work without interference. No significant logistical challenges were encountered, though scheduling steps such as flap harvest and pelvic dissection required deliberate sequencing to maintain workflow.

**FIGURE 2:**
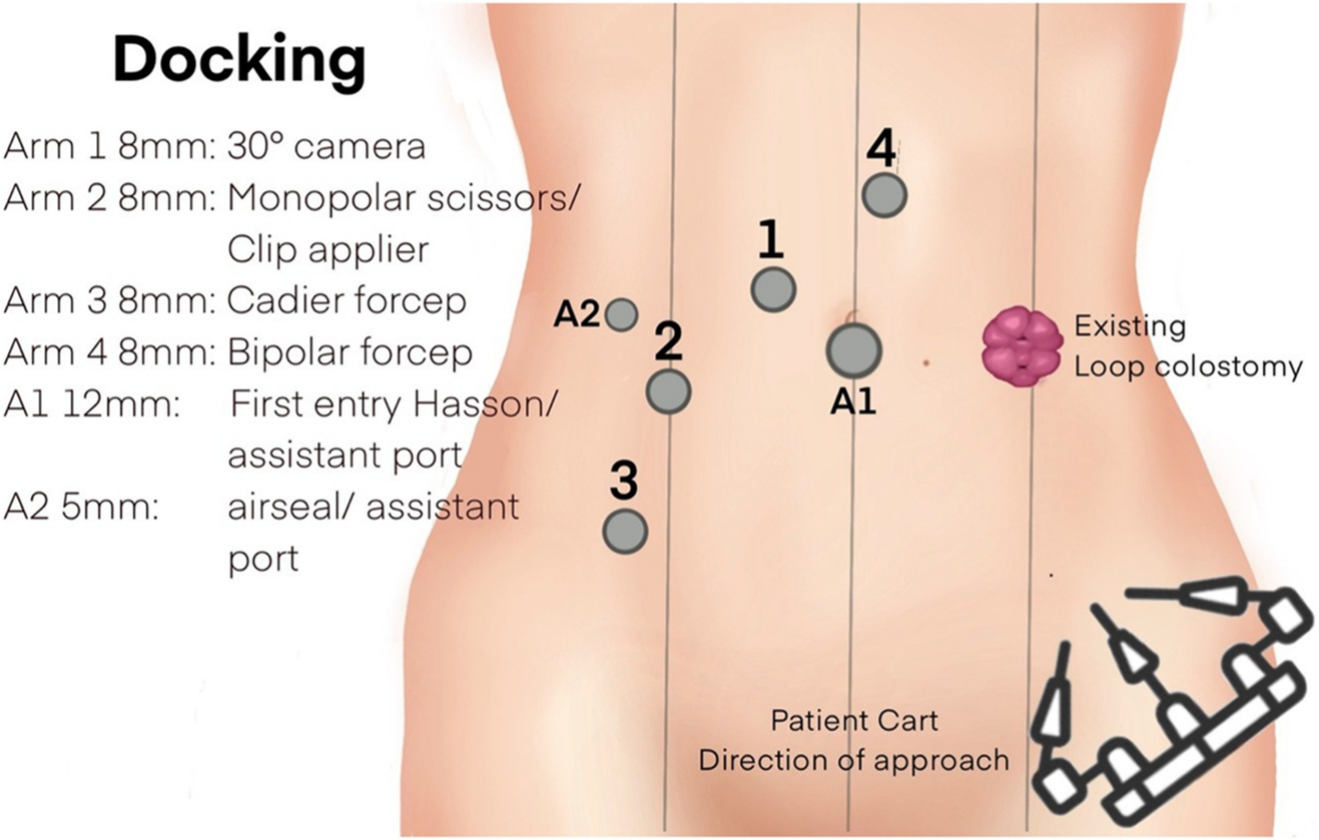
Robotic ports placement for beyond TME operation.

Perineal phase: Towards the end of the abdominal phase and after flap elevation, the space between the legs is free for the perineal dissection to be undertaken by the colorectal surgical team , and the specimen was retrieved en bloc (Figure 3).

**FIGURE 3:**
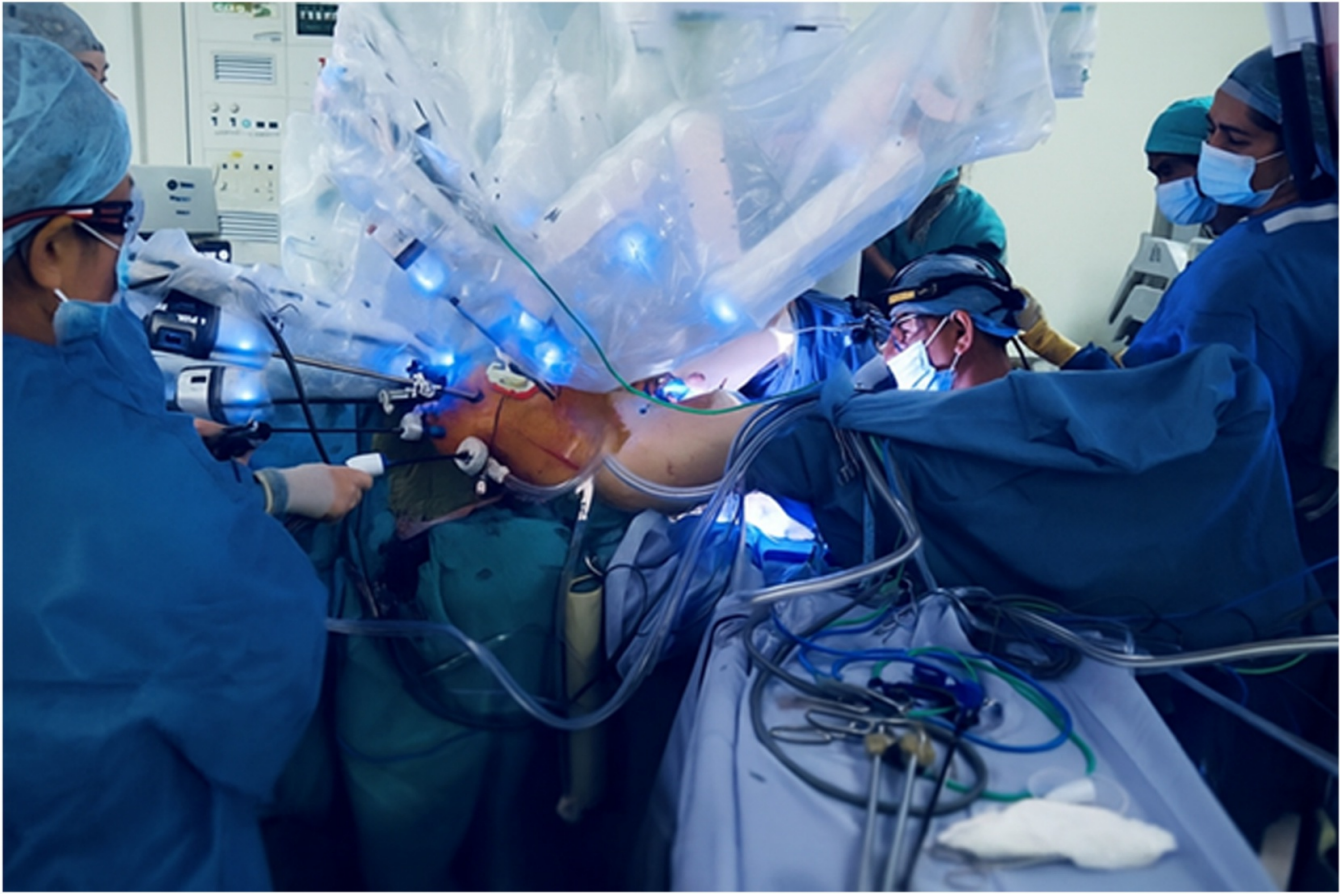
Final stage of abdominal phase with simultaneous pelvic phase.

Urological reconstruction and colostomy: In cases requiring urinary diversion, robotic dissection of the ileum and ureters was followed by extracorporeal uretero‐ileal anastomosis. The colorectal team fashioned the colostomy, removed ports under vision, and closed wounds. A transversus abdominus plane (TAP) block was administered under vision.

Plastic reconstruction: Once the perineal resection is complete and defect created, the gracilis flaps are retrieved from the tunnel over the posterior pubic ramus and double breasted in the pelvis to reconstruct the pelvic floor. If a PAP flap was required, this was inset at this time. This was performed in parallel with the final phases of urological and colorectal procedures such as colostomy or ileal conduit formation. If a pneumoperitoneum is required, once the gracilis flaps are inset there is enough seal to allow for inflation of the abdomen again.

### 2.5 Ethical Considerations

This study was exempted from ethical approval by the local hospital NHS Research Ethics Committee due to its retrospective design, use of anonymised clinical data and reliance on a prospectively maintained database without affecting standard patients' care. This study has been reported in accordance with the (PROCESS) guideline [16].

## 3 Results

Patient diagnosis, treatment, surgical intervention, hospital stay, and follow‐up timelines are summarised in Table 1.

**TABLE 1:** Summary timeline of cases.

Patient	Diagnosis	Neoadjuvant treatment	Surgical procedure	Flap type/conduit	Post‐operative complication	ICU LOS	Total LOS	90‐day readmission	90‐day mortality	Recurrence: Local/systemic
1	Rectal AC	Pre‐op CRT (TRG3)	TPE	Bilateral gracilis, ileal conduit	None	1	7	No	No	No recurrence
2	Anal SCC	Previous CRT	TPE	Bilateral gracilis, ileal conduit	Ileus (CD II)	0	18	No	Yes, cardiac arrest	—
3	Rectal AC	Pre‐op CRT (partial)	TPE	Bilateral gracilis, ileal conduit	Ileus (CD II)	0	14	Yes, pre‐sacral collection	No	No
4	Anal SCC	Previous RT	ELAPE, dissection inguinal lymph node, partial vaginectomy	Bilateral gracilis + PAP	Ileus, low Hb (CD II)	4	42	No	No	No
5	Anal SCC	Pre‐op RT	ELAPE	Bilateral gracilis	None	0	7	No	No	No
6	Anal SCC	Previous RT	ELAPE	Left gracilis + VY + ileal conduit	None	1	8	No	No	No
7	Rectal AC	None	PPE	Bilateral gracilis	Ileus (CD II)	0	14	Yes, pelvic collection	No	No/liver, gastric nodes metastasis
8	Rectal AC	Pre‐op CT (partial)	TPE	Left gracilis + IGAP + ileal conduit	Ileus + perineal infection (CD II)	1	31	Yes, pre‐sacral collection	No	No/liver metastasis, disease progression
9	Rectal AC	Pre‐op CRT (partial)	TPE	Bilateral gracilis	Atrial fibrillation (CD II)	0	15	No	No	No
10	Anal SCC	Previous RT	PPE	Bilateral gracilis + PAP	Partial flap dehiscence (CD II)	0	23	No	No	—

Abbreviations: AC, Adenocarcinoma; CD, Clavien Dindo; CRT, Chemoradiotherapy; CT, Chemotherapy; ELAPE, Extralevator Abdominoperineal excision; Hb, haemoglobin; ICU, Intensive care unit; IGAP, Inferior glutaeal artery perforator; LOS, Length of stay; PAP, Profunda artery perforator; PPE, Posterior pelvic exenteration; RT, radiotherapy; SCC, Squamous cell carcinoma; TPE, Total pelvic exenteration; TRG, Tumour Regression Grade.

### 3.1 Oncological Outcomes

Ten patients met the inclusion criteria. The mean age was 64 ± 9.3 years (range 50–80) and 60% were male. Fifty percent had an ASA grade of III, and mean BMI was 24.4 ± 3.5 kg/m^2^. One third were smokers (Table 2). Descriptive analysis was used, reporting mean ± standard deviation (SD) for normally distributed continuous variables, median and interquartile range (IQR) for skewed continuous variables, and proportions and percentage for categorical.

**TABLE 2:** Patient demographics and comorbidities.

Patient	Sex	Age	ASA	BMI (kg/m^2^)	PMH	Previous abdominal surgery
1	M	53	II	20	Hay fever	Nil
2	M	66	III	20.9	MI, DM, HTN	Laparoscopic loop colostomy
3	M	69	II	21	AF, pancreatic cancer	Whipple's resection
4	F	78	III	25	DM, coeliac disease	LAR & ileostomy
5	F	50	II	22.6	Non‐Hodgkin's lymphoma	Nil
6	M	80	III	27	HTN, prostate cancer	Nil
7	F	63	II	23.5	HTN	Nil
8	M	57	III	30.8	HTN	Appendicectomy
9	M	75	III	26	Hypothyroidism	Laparoscopic loop colostomy
10	F	50	I	27.7	Nil	Laparoscopic loop colostomy

Abbreviations: AF, Atrial Fibrillation; ASA, American Society of Anaesthesiologists; BMI, Body mass index; DM, Diabetes Mellitus; F, Female; HTN, Hypertension; LAR, Low anterior resection; M, Male; MI, Myocardial infarction; PMH, Past medical history.

Four patients presented with locally recurrent cancers (three anal, one rectal), all previously treated with curative intent and achieved R0 resections or complete clinical, pathological and radiological response. Treatment strategy varied based on tumour type and extent (Table 3): five patients underwent pelvic exenteration for advanced rectal adenocarcinomas (four total, one posterior), two underwent exenteration for anal cancer (one total, one posterior) and three underwent bTME ELAPE for low rectal or anal cancer.

**TABLE 3:** Pre‐operative oncologic characteristics.

Patient	Histological diagnosis	Surgery	Tumour location	Staging	CT/RT	Response
1	AC	TPE	Rectum	T3cN1	Pre op CRT	TRG3
2	SCC	TPE	Anal	N/A	Previous CRT	N/A
3	AC	TPE	Rectum	T4bN0M0	Pre op CRT	Partial
4	SCC	ELAPE	Anal	T4N0Mx	Previous RT	N/A
5	SCC	ELAPE	Anal	N/A	Pre op RT	N/A
6	SCC	ELAPE	Anal	T2N1aMx	Previous RT	N/A
7	AC	PPE	Rectum	T2N0M0	—	N/A
8	AC	TPE	Rectum	T4N1Mx	Pre op CT	Partial
9	AC	TPE	Rectum	T3N2M0	Pre op CRT	Partial
10	SCC	PPE	Anal	N/A	Previous RT	N/A

Abbreviations: AC, Adenocarcinoma; CRT, Chemoradiotherapy; CT, Chemotherapy; ELAPE, Extralevator Abdominoperineal excision; M, metastasis; N/A, not applicable; N, Node; PPE, Posterior pelvic exenteration; RT, radiotherapy; SCC, Squamous cell carcinoma; T, Tumour; TPE, Total pelvic exenteration; TRG, Tumour Regression Grade.

R0 resection was achieved in 80% of cases. Two patients (20%) had R1 resections due to threatened circumferential resection margins (CRM). One, with anal cancer, had a CRM of 0.4 mm but remained recurrence‐free at 2 years. The other patient developed systemic (liver and lung) metastases at 14 months without local recurrence. At 1‐year follow‐up, no patient experienced local recurrence; two developed systemic recurrence.

### 3.2 Operative Data

Median operative duration was 465 minutes (IQR 449–514, range 290–610). Robotic abdominal colorectal, urological abdominal, and perineal dissection phases averaged 125 ± 49, 127 ± 18, and 124 ± 54 minutes, respectively. Gracilis flap harvest overlapped with the robotic abdominal colorectal phase (mean 72 ± 5 minutes), while perineal flap reconstruction overlapped with the urological or stoma reconstruction phase (165 ± 37 minutes) (Table 4).

**TABLE 4:** Operative time breakdown (minutes).

Patient	Robotic abdominal phase with simultaneous gracilis flap harvest time		Urological abdominal time	Perineal dissection time	Flap reconstruction time (overlap final urology/stoma)	Total operative time
	Robotic phase	Gracilis phase				
1	95	66	135	90	170	460
2	132	80	120	122	132	465
3	97	66	150	110	150	445
4	207	71	N/A	130	236	525
5	98	74	N/A	100	101	290
6	215	68	113	95	160	530
7	101	67	99	125	180	465
8	63	76	140	265	195	610
9	135	77	135	70	176	480
10	110	75	N/A	135	145	340

Most flaps were bilateral Gracilis. Two patients required vaginal and pelvic reconstruction with a combined pedicled profunda artery perforator and bilateral Gracilis flaps. One patient had a V–Y flap in addition to a unilateral Gracilis flap. Median estimated blood loss (EBL) was 350 mL (IQR 200–600), while one patient exceeded 1 L due to severe radiation‐associated fibrosis. One patient required a lower midline incision to facilitate the pre‐sacral and pyriformis dissection (Table 5).

**TABLE 5:** Intraoperative details and flap reconstruction.

Patient	Organs removed	Flap reconstruction	EBL (mls)	Analgesia	Conversion to open	Reason for conversion
1	Sigmoid colon, rectum, levators, urinary bladder, prostate, anus	Bilateral gracilis flap, ileal conduit	300	PCA and epidural	N/A	—
2	Sigmoid colon, rectum, piriformis, right internal iliac vessels, urinary bladder, prostate, anus	Bilateral gracilis flap, ileal conduit	1450	PCA and epidural	Yes	To dissect the presacral tissues and pyriformis muscle
3	Rectum, levators, urinary bladder, anus	Bilateral gracilis flap, ileal conduit	200	PCA and epidural	N/A	—
4	Rectum, posterior vagina, puborectalis, anus, left groin nodes	Bilateral gracilis flap, PAP flap	800	PCA and epidural	N/A	—
5	Sigmoid colon, rectum, levators, anus	Bilateral gracilis flaps	400	PCA and spinal	N/A	—
6	Sigmoid colon, rectum, levators, urinary bladder, prostate, anus, left inguinal nodes	Left gracilis, V/Y flap. Ileal conduit	200	PCA	N/A	—
7	TAH & BSO, sigmoid colon, rectum, pyriformis, anterior vagina, anus	Bilateral gracilis flaps	200	PCA	N/A	—
8	Sigmoid colon, rectum, urinary bladder, coccyx, obturator internus, prostate, anus	Left gracilis, IGAP right side, ileal conduit	600	PCA and spinal	N/A	—
9	Sigmoid colon, rectum, coccyx, urinary bladder, prostate, levators, anus	Bilateral gracilis flaps	600	PCA and epidural	N/A	—
10	Sigmoid colon, rectum, levators, subtotal vaginectomy, anus	Bilateral gracilis flaps, PAP flap	200	PCA and epidural	N/A	—

Abbreviations: BSO, Bilateral salpingo‐oophorectomy; EBL, Estimated blood loss; IGAP, Inferior glutaeal artery perforator; N/A, not applicable; PAP, Profunda artery perforator; PCA, Patient controlled analgesia; TAH, Total abdominal hysterectomy.

### 3.3 Complications and Follow‐Up

Postoperative outcomes were favourable, with no reoperations. The most common 30‐day complication was ileus (50%). Three patients were re‐admitted within 90 days with collection: two were conservatively managed with antibiotics and one required image‐guided drainage. One patient experienced sudden cardiac death within 90‐day. Median length of stay (LOS) was 14.5 days (IQR 9.5–21.8, range 7–42). 90‐day readmission and mortality rates were 30% and 10%, respectively.

Follow‐up was conducted in accordance with national guidelines, including clinical assessment and CEA every 3–6 months, and CT/pelvic MRI every 6 months during the first 2 years. Surveillance focused on early detection of recurrence, flap viability, stoma function, and perineal wound healing. A multidisciplinary team approach was maintained, with follow‐up planned for up to 5 years.

## 4 Discussion

This study presents the initial outcomes of robotic bTME combined with synchronous flap reconstruction of the perineum for primary or recurrent locally advanced rectal and anal cancers. The results highlight that a minimally invasive robotic approach is feasible for these complex operations, and that synchronous gracilis flap reconstruction can reduce operative duration in long procedures. Robotic pelvic exenterations were initially reported for bladder and cervical cancers in 2007 and 2009, with application to advanced rectal cancer introduced in 2014 [8, 17, 18].

Prior studies describe mean total operative times for robotic pelvic exenterations ranging from 534 to 612 minutes [8, 19]. In comparison, our median total operative time was 465 minutes (IQR 448–513), including flap reconstruction. This reduction reflects effective collaboration between colorectal and plastic surgery teams, facilitated by simultaneous gracilis flap harvesting during the robotic abdominal phase (median of 72 minutes, IQR 67–75), optimising theatre efficiency without compromising safety.

Regarding morbidity, Piozzi et al. and Saqib et al. reported major complication rates of 19.7% and 30.7%, respectively, in robotic pelvic resections with mean operative times between 360 and 450 minutes [20, 21]. Our study showed no Clavien‐Dindo grade III complications within 30 days, suggesting that robotic bTME with synchronous gracilis flap reconstruction may reduce high‐grade complications, though larger studies are needed to confirm this observation.

Perineal reconstruction is critical in extensive resections, with various techniques described including rectus, glutaeus, and gracilis flaps, as well as biological mesh [22, 23, 24]. Our institution favours the gracilis muscle flap, first described in 1972, owing to its reliability, technical simplicity, and ability to reconstruct large perineal defects while maintaining its integrity [25, 26, 27]. The muscle gives an anatomical reconstruction of the levator ani complex being slung from the pubic ramus anteriorly to sacrum posteriorly and pelvic side wall laterally. It brings healthy unirradiated muscle into the defect aiding wound healing and reducing dead space. Even bilateral gracilis flaps does not fill as much dead space as a large Vertical rectus abdominus myocutaneous (VRAM) flap, but they do not lead to such a major muscle defect and can lead to abdominal wall hernias the VRAM can [28, 29]. The gracilis flap offers minimal donor site morbidity, concealed scars, and effective pelvic floor reconstruction without requiring mesh. Moreover, the minimally invasive abdominal approach and no VRAM donor site allows for all options of multiple stoma site placement [15]. Their use also negates the increased time and morbidity associated with turning the patient prone for gluteal flaps. A recent study evaluating 40 patients who underwent synchronous gracilis flap reconstruction reported low donor site morbidity (12.5%), acceptable recipient site complications, no 30‐day mortality, and over 92% survival at 2 years, concluding that the gracilis flap provides outcomes comparable to abdominal‐based flaps with fewer donor site complications [30]. These findings support the gracilis flap as a primary reconstructive option in appropriate cases.

Biological mesh use has been associated with wound complication rates comparable to primary closure and flap reconstruction, alongside similar costs [31, 32, 33]. In our series, one patient (10%) developed perineal wound infection managed conservatively, and one (10%) experienced partial cutaneous flap dehiscence managed with vacuum‐assisted closure, with no flap loss, reinforcing the reliability of the gracilis flap.

Synchronous gracilis flap reconstruction while the robotic abdominal phase is ongoing optimises operative efficiency and minimises anaesthesia time.

Evidence in literature suggest a reduced conversion rate with robotic surgery, typically 5%–10% [34, 35]. In our study, one patient (10%) required a planned lower midline incision following complete robotic mobilisation to facilitate the presacral and pyriformis dissection. Despite advances in minimally invasive approaches, bTME remains complex with significant morbidity [4, 36]. Oncological outcomes vary; Shin et al. reported a 93% R0 resection rate and comparable local recurrence at 2 years, highlighting the importance of clear margins for survival [8, 37]. Our series achieved 80% R0 resections. Although robotic TME offers comparable oncological outcomes to laparoscopy, recent data suggest additional benefits in technically challenging cases, such as patients with obesity, male gender, low‐lying tumours, prior neoadjuvant chemoradiotherapy and a narrow pelvis. Large multicentre and single‐surgeon series of robotic TME have demonstrated comparable circumferential resection margin involvement, disease‐free survival, and local recurrence rates, along with improved mesorectal excision quality and reduced conversion rates. Such technical advantages may be particularly valuable during bTME, where precise dissection within a previously treated or anatomically constrained pelvis is often required. Our experience supports the feasibility of this approach in patients with complex primary and recurrent pelvic malignancies [38, 39, 40]. In such high‐risk groups, the PelvEx Collaborative reported a 37.8% incidence of Clavien‐Dindo grade III–IV complications and a 1.5% 30‐day mortality, reflecting the complexity of these surgeries [41].

Limitations of our study include the small sample size, retrospective single‐centre design, absence of a control group, heterogeneity of surgical procedures, and short follow‐up period. Learning curve effects and institutional expertise may have influenced outcomes, limiting generalisability. Nonetheless, our findings provide a foundation for future research. Multi‐centre studies with larger cohorts and prospective designs are needed to validate the oncological safety, long‐term outcome, and feasibility of robotic bTME. Future studies should also explore patient‐reported functional outcomes, quality of life, and cost‐effectiveness of different reconstructive approaches. Despite these limitations, our results demonstrate that robotic bTME with synchronous gracilis flap reconstruction is feasible, safe, and effective in reducing operative durations for high‐risk pelvic surgeries.

## 5 Conclusion

Robotic bTME with synchronous gracilis flap reconstruction of the perineum for locally advanced primary or recurrent ano‐rectal cancer is safe, feasible and technically achievable with an acceptable morbidity. We believe that this may reduce theatre time and is more palatable for staff members as the operation finishes in a timely fashion. Future research should focus on validating these findings in larger prospective studies to establish the role of robotic bTME with synchronous flap reconstruction as a standard approach for complex anorectal cancers.

## Author Contributions

M.R.I./A.H.A./I.A.S./A.R.M./R.H.: Study concept and design, data collection, data analysis, writing – reviewing and editing. DD: Data collection, drafting figures. E.J.M.R./SK: Reviewing. I.A.S.: Corresponding author.

## Funding

The authors have nothing to report.

## Ethics Statement

Exempted according to local NHS Research Ethics Committee guidelines due to the nature of the study.

## Conflicts of Interest

Irshad A. Shaikh is a European proctor for Intuitive.

## Data Availability

The data that support the findings of this study are available from the corresponding author upon reasonable request.
